# Supplementary material for: Akkermansia, a Possible Microbial Marker for Poor Glycemic Control in Qataris Children Consuming Arabic Diet—A Pilot Study on Pediatric T1DM in Qatar
Source: Nutrients. 2021 Mar 4;13(3):836. doi: 10.3390/nu13030836 (PMC7999932; doi:10.3390/nu13030836)

Supplementary Figure S1. Shows the gut microbial relative abundance of the study participants (n=28) at phyla level.

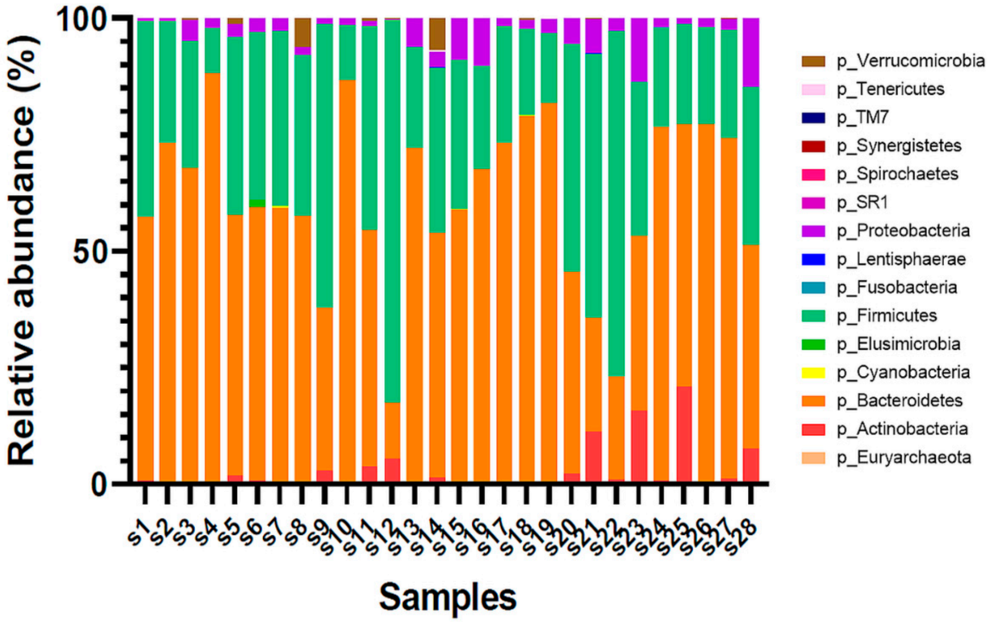

Supplementary Figure S2. Shows the relative abundance of genus *Akkermansia* in samples which are classified at different groups. Samples are classified based on (a) CSII therapy, (b) low or high HbA1C level, (c) CSII therapy along with the diet pattern, (d) low or high HbA1C level along with the diet pattern, (e) expatriate or Qatari subjects along with the CSII therapy, (f) expatriate or Qatari subjects along with low or high HbA1C level, (g) expatriate or Qatari subjects, (h) subjects with either Arabic_diet or mixed_diet pattern, and (i) expatriate or Qatari subjects along with the either Arabic_diet or mixed_diet pattern. Statistical significance was calculated using Kruskal-Wallis test and Mann Whittney test, where applicable. * $p<0.05$, ** $p<0.01$, and *** $p<0.001$. (j) Shows the relative abundance of *Akkermansia* in T1DM subjects compared with HC subjects. T1DM, n=28; HC, n=7.

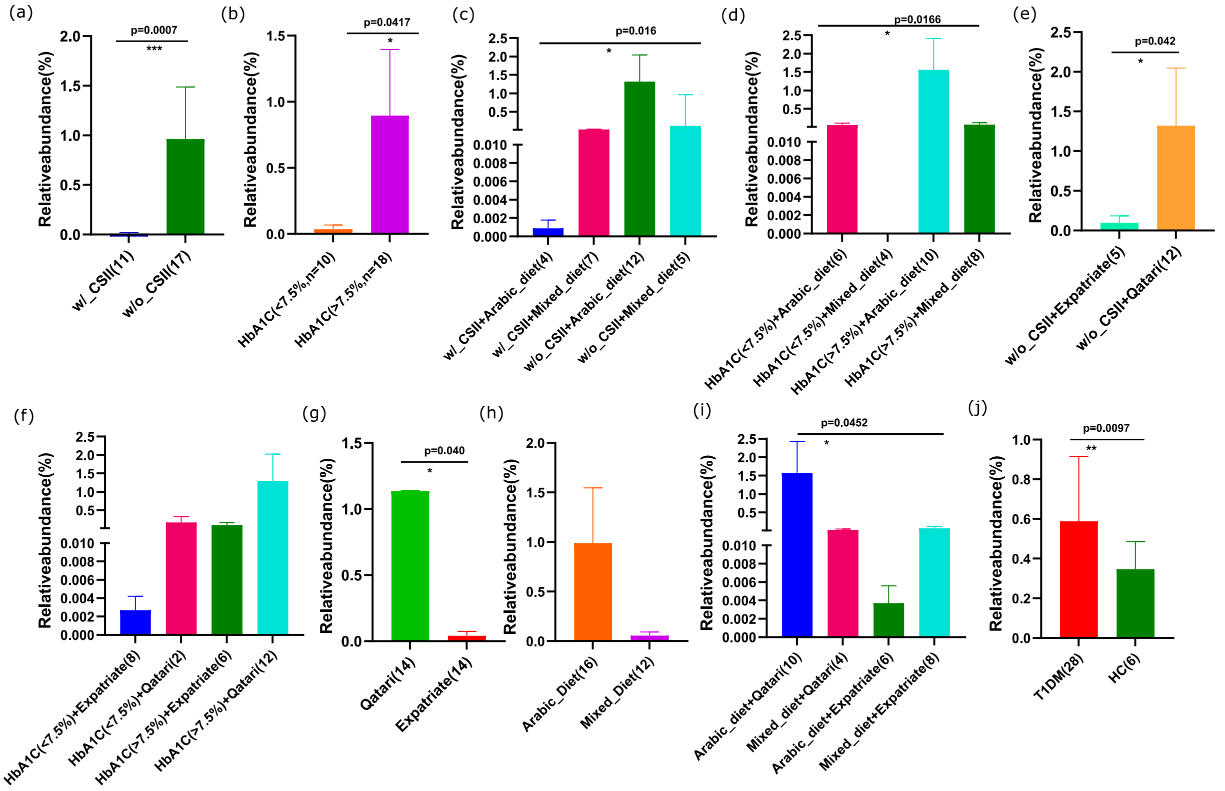

Supplement: Supplementary file 1 [file nutrients-13-00836-s001.pdf]
